# Brain White Matter Shape Changes in Amyotrophic Lateral Sclerosis (ALS): A Fractal Dimension Study

**DOI:** 10.1371/journal.pone.0073614

**Published:** 2013-09-09

**Authors:** Venkateswaran Rajagopalan, Zao Liu, Didier Allexandre, Luduan Zhang, Xiao-Feng Wang, Erik P. Pioro, Guang H. Yue

**Affiliations:** 1 Department of Biomedical Engineering, Lerner Research Institute, Cleveland Clinic, Cleveland, Ohio, United States of America; 2 Human Performance and Engineering Laboratory, Kessler Foundation Research Center, West Orange, New Jersey, United States of America; 3 Department of Quantitative Health Sciences, Cleveland Clinic, Cleveland, Ohio, United States of America; 4 Neuromuscular Center and Department of Neurology, Neurological Institute, Cleveland Clinic, Cleveland, Ohio, United States of America; 5 Department of Neurosciences, Lerner Research Institute, Cleveland Clinic, Cleveland, Ohio, United States of America; 6 Department of Physical Medicine & Rehabilitation, University of Medicine and Dentistry of New Jersey, Newark, New Jersey, United States of America; Baylor College of Medicine, Jiao Tong University School of Medicine, United States of America

## Abstract

Amyotrophic lateral sclerosis (ALS) is a fatal progressive neurodegenerative disorder. Current diagnosis time is about 12-months due to lack of objective methods. Previous brain white matter voxel based morphometry (VBM) studies in ALS reported inconsistent results. Fractal dimension (FD) has successfully been used to quantify brain WM shape complexity in various neurological disorders and aging, but not yet studied in ALS. Therefore, we investigated WM morphometric changes using FD analyses in ALS patients with different clinical phenotypes. We hypothesized that FD would better capture clinical features of the WM morphometry in different ALS phenotypes than VBM analysis. High resolution MRI T1-weighted images were acquired in controls (n = 11), and ALS patients (n = 89). ALS patients were assigned into four subgroups based on their clinical phenotypes.VBM analysis was carried out using SPM8. FD values were estimated for brain WM skeleton, surface and general structure in both controls and ALS patients using our previously published algorithm. No significant VBM WM changes were observed between controls and ALS patients and among the ALS subgroups. In contrast, significant (p<0.05) FD reductions in skeleton and general structure were observed between ALS with dementia and other ALS subgroups. No significant differences in any of the FD measures were observed between control and ALS patients. FD correlated significantly with revised ALS functional rating scale (ALSFRS-R) score a clinical measure of function. Results suggest that brain WM shape complexity is more sensitive to ALS disease process when compared to volumetric VBM analysis and FD changes are dependent on the ALS phenotype. Correlation between FD and clinical measures suggests that FD could potentially serve as a biomarker of ALS pathophysiology, especially after confirmation by longitudinal studies.

## Introduction

Amyotrophic lateral sclerosis (ALS) is a progressive neurodegenerative disease that affects both upper motor neurons (UMNs) and lower motor neurons (LMNs). Diagnosis of ALS is based on both UMN and LMN degeneration signs. Electromyography (EMG) provides an objective method to estimate the LMN involvement even though the LMN dysfunction cannot be observed clinically. However, no equivalent method exists to detect UMN involvement [1]–[3]. Because no specific test exists to definitively diagnose ALS, diagnosis is based on identifying consistent clinical features and laboratory investigations (e.g., blood tests, EMG, and neuroimaging) to exclude other conditions that mimic ALS [4]. This usually results in significant delay before a definitive diagnosis is made, averaging ∼12 months from symptom onset. Given that ∼80% of patients survive an average of 3–5 years from symptom onset [5], shortening the time to correct diagnosis is imperative. There has been great interest in identifying biomarkers of ALS, which would allow earlier diagnosis, monitoring disease progression and assessing recognition of efficacy of pharmacotherapies.

MRI, a noninvasive technique, is best suited for diagnosis of UMN involvement and has shown various brain abnormalities in ALS. Conventional MRI using T2-, proton density-, and fluid attenuated inversion recovery (FLAIR)-weighted sequences usually show no visible abnormalities in ALS brains. However, in some ALS patients with dementia (especially in condition affecting frontotemporal lobe of the brain) and at an advanced stage of the disease, atrophy in brain grey and white matter structures is evident in their MR images. Also, between 17% and 67% (median 40%) of ALS patients display hyperintensity of the bilateral corticospinal tract (CST) in conventional T2- and proton density-weighted images [6]. Based on one radiologic-pathologic study, such hyperintensity represents demyelination and Wallerian degeneration of the descending CST fibers [7]. Most previous MRI brain studies in ALS have identified such CST hyperintensity qualitatively (i.e. relying on visual evaluation) [6], which is prone to error. These various brain anomalies suggest that pathological mechanisms of ALS may be different among ALS patients. Therefore, quantitative assessment of brain MRI information in ALS should be made more objective to better assess varying brain abnormalities among the different ALS phenotypes.

At microscopic levels ALS is characterized by axonal swelling with neurofilament accumulations, axonal Wallerian degeneration and dendrites attenuation [8]. The microscopic changes such as axon degeneration [9] and demyelination may in turn lead to changes in macroscopic level. If it is the case, a reduction in the complexity of the WM structure is expected to occur, which could possibly serve as a biomarker for detecting degenerative changes in the brain brought out by ALS disease process.

Volumetric analysis based on VBM is one of the most commonly used methods to quantify structural changes. However previous VBM studies on brain WM in ALS have reported inconsistent results. Some studies [10], [11] showed significant WM volume changes in ALS patients when compared to controls while others did not [12], [13]. Such discrepancies may be due to factors such as differences in methodology or masking potential effects by combining ALS patients with differing phenotypes averaging out differences that may have been detected otherwise. Looking at differences at the voxel level throughout the whole brain constitutes the main advantage but also a major limitation of the VBM technique as it imposes strict statistical constraints to adjust for multiple comparisons, potentially failing to reach statistical significance. Furthermore VBM only estimate WM atrophy and is not sensitive to other structural morphometric features such as shape. Fractal dimension (FD) is another quantitative approach that addresses some of VBM’s limitations by providing a global measure of internal shape complexity of brain WM. FD have been successfully used to study brain WM adaptations in aging [3], [14] and in various neurological disorders [15], [16] but has yet to be applied to study ALS.

Therefore, the aim of this study was to investigate WM structural degeneration in ALS patients with different clinical phenotypes by quantifying shape morphometric changes using FD method and comparing results to VBM. Since FD analysis is a sensitive measure of shape (complexity) changes when compared to VBM [3] we hypothesized that a) FD analysis would be more sensitive to detect differences between ALS patients and controls than the VBM approach and b) FD analysis will bring out significant differences between ALS subgroups.

## Methods

### Ethics Statement

The Cleveland Clinic Institutional Review Board approved the study and waived the need for informed consent from participants.

### Demographics

A total of 100 subject’s data collected as part of our routine clinical scan were assigned into either control or one of the four ALS patient subgroups based on their clinical signs and clinical evaluation of their MRI images: (1) neurological controls, (2) ALS patients with frontotemporal dementia (ALS-FTD), (3) upper motor neuron (UMN)-predominant ALS patients with corticospinal tract (CST) hyperintensity on T2/PD-weighted images (ALS-CST+), (4) UMN-predominant ALS patients without CST hyperintensity identified on T2/PD-weighted images (ALS-CST–), and (5) ALS-classic patients (ALS-Cl). UMN-predominant ALS patients were defined as those with either no lower motor neurons signs or if present, were restricted to only one neuraxial level (bulbar, cervical, or lumbosacral) at the time of MRI. UMN patients with CST hyperintensity were those in whom hyperintense signal was observed along the CST in T2- and PD-weighted images. ALS patients with frontotemporal dysfunction or dementia (ALS-FTD) were identified by clinical (e.g., Montreal cognitive assessment score <26) or formal neuropsychometric testing. Demographics of the above patient population and clinical measures disease duration, ALSFRS-R score, El Escorial score (EES) and disease progression rate of ALS patients are given in Table 1. Disease progression rate is calculated as given in equation 1 below [17]–[20]. EES being ordinal was converted from a category measure into a numeric form as follows: possible ALS (EES = 1), probable with laboratory support (EES = 2), probable (EES = 3), and definite (EES = 4).
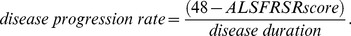
(1)


**Table 1:** Participants’ characteristics.

		All	Control	ALS-FTD	ALS-CST+	ALS-CST–	ALS-Cl	Anova/Chi-Square
N		97	11	20	20	24	22	
**Demographics**								
Age		57.9±12.7	51.7±16.6^a^	66.7±9.9^a, b, c, d^	52.9±11.5^b^	58±11.5^c^	57.4±11.5^d^	0.003
Gender								
Male		54 (56%)	8 (73%)	7 (35%)	13 (65%)	14 (58%)	12 (55%)	0.24
Female		43 (44%)	3 (27%)	13 (65%)	7 (35%)	10 (42%)	10 (45%)	
**Disease Characteristics**								
ALSFRS-R		34.6±8.4		30.5±7.2^a^	34.9±8	35±8.6	37.2±8.7^a^	0.10
Disease Duration		19 [12; 48]		34 [18.8; 45]	13 [9.5; 18.3]	35.5 [17.8; 55]	13 [9; 52]	
Ln		3.13±0.93		3.47±0.62^ a,b^	2.45±0.67^ a, c^	3.59±1.02^ c, d^	2.92±1.02^ b, d^	0.0001
Progression Rate		0.54 [0.24; 0.83]		0.54 [0.34; 0.73]	0.81 [0.53; 1.17]	0.32 [0.16; 0.71]	0.5 [0.19; 0.85]	
Ln		−0.77±1.07		−0.71±0.61 ^a^	−0.05±0.83 ^a, b, c^	−1.3±1.23 ^b^	−0.85±1.05^ c^	0.002
EES Score								
1		38 (45%)		7 (35%)	10 (50%)	18 (75%)	3 (15%)	0.02
2		14 (17%)		2 (10%)	4 (20%)	2 (8%)	6 (30%)	
3		23 (27%)		7 (35%)	5 (25%)	3 (13%)	8 (40%)	
4		9 (11%)		4 (20%)	1 (5%)	1 (4%)	3 (15%)	

N = Number of subjects, Ln = natural log transform. EES score: 1 = defined as possible ALS, 2 = probable with laboratory support. 3 = probable and 4 = definite. Please refer to the Method section for the definition of ALS-FTD, ALS-CST+, ALS-CST–, ALS-Cl. Data are represented as N (percentage) for non categorical data, Mean ± Standard Deviation for normally distributed data, and Median [25^th^; 75^th^ percentile] for non normally distributed data. Statistical analysis was performed on the natural log transform for disease duration and Disease Progression Rate to obtain normally distributed data.

Disease characteristics were not available for all subjects. Sample size for ALS-FTD/ALS-CST+/ALS-CST−/ALS-Cl are N = 17/18/23/20 for ALSFRS-R, N = 20/20/24/21 for disease duration, N = 17/18/23/19 for Disease progression and N = 20/20/24/20 for EES score.

Group connected by the same superscript letters for each outcome are significantly different (p<0.05) based on student t-test. For example, Control and ALS-FTD connected via the letter “a” are significantly different in age.

### Imaging Protocol

High resolution 3D T1-weighted axial MRI images of the whole brain were obtained using magnetization prepared rapid gradient echo (MPRAGE) sequence using 1.5T Siemens Symphony (Erlangen, Germany) scanner. Imaging parameters were: TR (repetition time) = 1800 ms, TE (echo time) = 4.38 ms, flip angle = 10°, inversion time (TI) = 1100 ms, slice thickness = 1 mm, in-plane resolution = 0.9×0.9 mm^2^, and number of slices = 160. T_2_- and PD-weighted images were also obtained as part of our clinical protocol using a dual-echo FSE sequence; the imaging parameters were: TR = 3900 ms, TE = 26 ms and 104 ms, echo train length or turbo factor = 7, number of averages = 1, slice thickness = 4 mm, in-plane resolution = 0.9 × 0.9 mm, and a total of 40 contiguous slices.

### Data Processing

PD and T2-weighted images were primarily used to identify CST hyperintensity, as part of the routine clinical evaluation of ALS patients. WM morphometric changes in T1-weighted images between control and ALS patients were studied using VBM and FD approaches.

### White Matter VBM Analysis

White matter VBM analysis was carried out using SPM8 software (http://www.fil.ion.ucl.ac.uk/spm/) [21]. VBM8 batch processing modules were used to perform WM VBM analysis. First step ‘estimate and write’ involved correcting for inhomogeneities, brain extraction, and segmentation of brain into GM, WM and CSF volume probability maps. A study specific template was then created from a subgroup of patients drawn from the study population randomly using ‘DARTEL create template’ module [22]. All the subjects’ WM volumes were then nonlinearly registered to the specific template using ‘DARTEL existing template’ module. These images were then brought to MNI space using ‘Normalize to MNI space module’. The resulting images were then smoothed using a Gaussian kernel of 3 mm with a full-width half-maximum of ∼7 mm. Finally, the smoothed images were subjected to statistical inference using non-parametric approach using (FSL ‘randomise’ (since non-parametric toolbox in SPM i.e. SnPM cannot accommodate for regressing out age and clinical measures) based on general linear model to identify voxel-wise differences in WM volume between the control and ALS subgroups, and among ALS subgroups regressing out age, disease duration and ALSFRS-R score. A p<0.05 corrected for multiple comparisons using family-wise error rate was considered the level of significance.

### White Matter Fractal Dimension (FD) Analysis

FD analysis was carried out using our customized in-house routines (details described elsewhere) [3]. Briefly the image processing included: skull stripping of T1-weighted images using FMRIB Software Library (FSL) Brain Extraction Tool [23] (http://www.fmrib.ox.ac.uk/fsl/Center for Functional Magnetic Resonance Imaging of the Brain Oxford, UK). Brain extraction was followed by segmentation into WM, grey matter (GM) and cerebrospinal fluid probability maps using FSL’s FAST tool [24]. WM probability maps were then binarized using a threshold value of 0.5. A 3D thinning method was then applied to the WM binary image in order to obtain the 3D WM skeleton. The 3D thinning algorithm removed as many boundary voxels as possible without changing the general shape of the WM, until a center line of one voxel width (skeleton) remained. Left and right hemispheres were then separated from the whole brain using FSL tools. Masks of left and right hemispheres separated in the previous step were applied to WM skeleton and WM general structure images to get the WM skeleton and WM general structure of left and right hemispheres. FD values were estimated using a 3D box-counting method (details described elsewhere) [25]. The box-counting method was preferred since it can be applied to structures without self-similarity, such as the human brain. (The box-counting method works by repeatedly applying different-sized meshes (*r*) to the fractal image and counting the number of boxes (*N*) needed to completely cover the fractal.) Finally, a linear regression fit after log transformation was used to estimate FD values using equation 2 given below
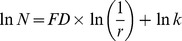
(2)where *k* is a nuisance parameter, in self-similar scale (linear portion in the logarithmic function).

In this study we estimated FD values of the three WM features (shape representations): skeleton, surface and general structure. Skeleton FD was calculated by counting the boxes needed to cover the WM skeleton; surface FD was evaluated by counting the boxes needed to cover the boundary of WM/GM interface; general structure FD was estimated by counting the boxes needed to cover all the WM voxels (which included skeleton and surface). The skeleton (consists of central line of each WM tract/bundle), also known as WM interior structure that preserves the topological and geometric information of the WM. The skeleton configuration represents the interior structure complexity of the brain WM. The surface structure consists of voxels at the boundary i.e. GM/WM interface, reflecting the shape of the gyral and sulcal convolutions in the GM/WM interface. General structure comprises of all WM voxels (including voxels in the GM/WM boundary and skeleton) in WM segmented images, representing the volume changes. Because the WM skeleton, general structure and surface represent three different aspects of brain WM structure, it was expected that they may serve as more comprehensive and distinct shape complexity measures to evaluate the WM structure shape/structure changes brought out by ALS disease process than other approaches.

### Statistical Method

Between group comparison of participants’ demographics and patients clinical measures were first performed to check for potential group differences using ANOVA for age, ALSFRS-R and disease duration and Pearson chi-square for gender and EES. Disease duration and progression rate were natural log transformed to satisfy normality requirement of ANOVA.

Statistical comparisons of FD values of all the three features of WM structures (skeleton, surface and general structure) between control and ALS subgroups, and among the ALS subgroups were carried out using linear mixed-effect regression models [26]. An outlier value was detected through outlier and leverage-point diagnostics method, and was eliminated from further analysis. Previous studies have shown reduction/changes in FD values with age^3.^ Also, since we observed significant age differences between our patient groups (Table 1), age was added as a covariate in our statistical model to eliminate its confounding effect. Thus, the expected value of the response (i.e. each of the three WM structure features) was modeled as a linear function of the age and group. To control the heterogeneity of data, a gender-specific random effect was also included in the model. The restricted maximum likelihood estimation method was used to fit the models we specified. Subsequent pairwise comparisons between groups were performed based on the fitted models [27]. Tukey's honestly significant difference test was applied for the multiple comparisons in order to control the probability of making a type I error in the multiple testing problem [28].

Given the marginal group difference in the ALSFRS-R score and strong correlation with FD, we also performed a sensitivity analysis to assess how this difference could potentially explain group difference in FD. To this purpose, we reran the mixed model on all ALS patients (excluding control) with ALSFRS-R score added as a fixed effect covariate. Despite group differences observed for disease duration and disease progression rate, they fail to fit the linear regression model and were not included in the model.

To assess the relation between clinical measures and FD values, ALSFRS-R, disease duration and disease progression rate were correlated with FD values of general structure (whole brain, left and right hemisphere), surface (whole brain, left and right hemisphere) and skeleton (whole brain, left and right hemisphere) using Spearman’s correlation analysis. El Escorial score (EES) being ordinal, was converted from a category measure into a numeric form as “El Escorial score” (EES): possible ALS (EES = 1), probable with laboratory support (EES = 2), probable (EES = 3), and definite (EES = 4). ALS patients were then re-grouped based on their EES and were assigned to one of the above four groups. FD values of patients with different El Escorial scores were then compared using Kruskal-Wallis test with Bonferroni correction.

## Results

### Participants’ Demographics and Patients’ Disease Characteristics

As shown in Table 1, significant group differences were found for age (p = 0.003), disease duration (p = 0.0001), disease progression rate (p = 0.002) and EES score (p = 0.02). Marginal difference was also found for ALSFRS-R score.

Overall ALS-CST+ and controls were younger than all other groups. Post-hoc analysis using student t-test revealed that ALS-FTD were significantly older than control (p = 0.001), ALS-CST+ (p = 0.0004), ALS-CST– (p = 0.02) and ALS-Cl (p = 0.01). For participants’ demographics and disease characteristics, values were not corrected for multiple comparisons to protect from possible false negatives.

Similarly disease duration was significantly greater for ALS-FTD and ALS-CST– compared to ALS-CST+ (p<0.001) and ALS-Cl (p = 0.04 and p = 0.008 respectively). Disease progression rate was significantly faster for ALS-CST+ than ALS-CST– (p = 0.0001), ALS-Cl (p = 0.02) and ALS-FTD (p = 0.05). Disease progression for ALS-CST– was marginally slower than ALS-FTD (p = 0.07).

ALSFRS-R is a standardized measure, widely used for evaluating functional status of ALS patients. Even though only a marginal group difference was observed for ALSFRS-R (p = 0.10), given its strong correlation with FD (see end of this section), we performed a post-hoc analysis revealing that ALS-FTD had a lower score which was significantly different than ALS-Cl (p = 0.02) and marginally different than ALS-CST– (p = 0.09).

A significant group effect was also observed for the EES score (p = 0.02) with a greater percentage of patients classified as possible ALS (EES = 1) and probable ALS with laboratory support (EES = 2) for ALS-CST+ (80%) and ALS-CST– (83%) compared to ALS-FTD and ALS-Cl (45%).

### White Matter VBM

WM VBM analysis failed to reveal any significant difference either between control and one of the four ALS subgroups or among the subgroups. No significant correlation was observed between WM volume and the clinical measures ALSFRS-R, disease duration or disease progression rate. No significant correlation was found between patient groups with EESs of possible, probable with lab support, probable and definite ALS patients.

### FD WM Structural Complexity

On the other hand, FD of skeleton and general structure revealed significant group effect differences between ALS-FTD and ALS-CST+ groups after correcting for multiple comparisons. Mean and standard deviation values of FD WM skeleton, surface and general structure of the whole brain, left and right hemisphere WMs in all five groups and corresponding *P* value of age effect and group effect are given in Tables 2 and 3.

**Table 2:** White matter fractal dimension values.

Region	Control (M±SD)	ALS-FTD(M±SD)	ALS-CST+(M±SD)	ALS-CST–(M±SD)	ALS-Cl(M±SD)
*Skeleton*					
LH	2.407±0.018	2.404±0.030	2.420±0.022	2.406±0.027	2.412±0.022
RH	2.409±0.021	2.394±0.023	2.420±0.021	2.400±0.025	2.403±0.019
WB	2.487±0.018	2.469±0.020	2.501±0.024	2.480±0.023	2.484±0.017
*Surface*					
LH	2.462±0.018	2.468±0.020	2.467±0.015	2.464±0.019	2.471±0.013
RH	2.467±0.024	2.465±0.017	2.472±0.013	2.465±0.022	2.468±0.014
WB	2.549±0.017	2.551±0.020	2.557±0.013	2.547±0.019	2.557±0.014
*General Structure*					
LH	2.582±0.054	2.572±0.044	2.590±0.035	2.582±0.044	2.590±0.041
RH	2.598±0.020	2.583±0.016	2.603±0.033	2.575±0.048	2.581±0.050
WB	2.633±0.013	2.618±0.013	2.638±0.015	2.625±0.015	2.629±0.015

M±SD = Mean = 0.015rd Deviation, LH = Left hemisphere, RH = Right hemisphere, WB = Whole brain, ALS-FTD = ALS patients with frontotemporal dementia, ALS-CST+ = ALS patients with CST hyperintensity, ALS-CST– = ALS patients without CST hyperintensity, ALS-Cl = classic ALS.

**Table 3:** *P* value of age effect and group effect.

Region	*P* value	
	Age	Group
*Skeleton*		
LH	0.06	0.54
RH	0.25	0.05*
WB	0.24	0.003*
*Surface*		
LH	0.30	0.71
RH	0.42	0.80
WB	0.64	0.34
*General Structure*		
LH	0.81	0.67
RH	0.19	0.29
WB	0.0001*	0.05*

LH = Left hemisphere, RH = Right hemisphere, WB = Whole brain.

* P<0.05.

**Figure 1:**
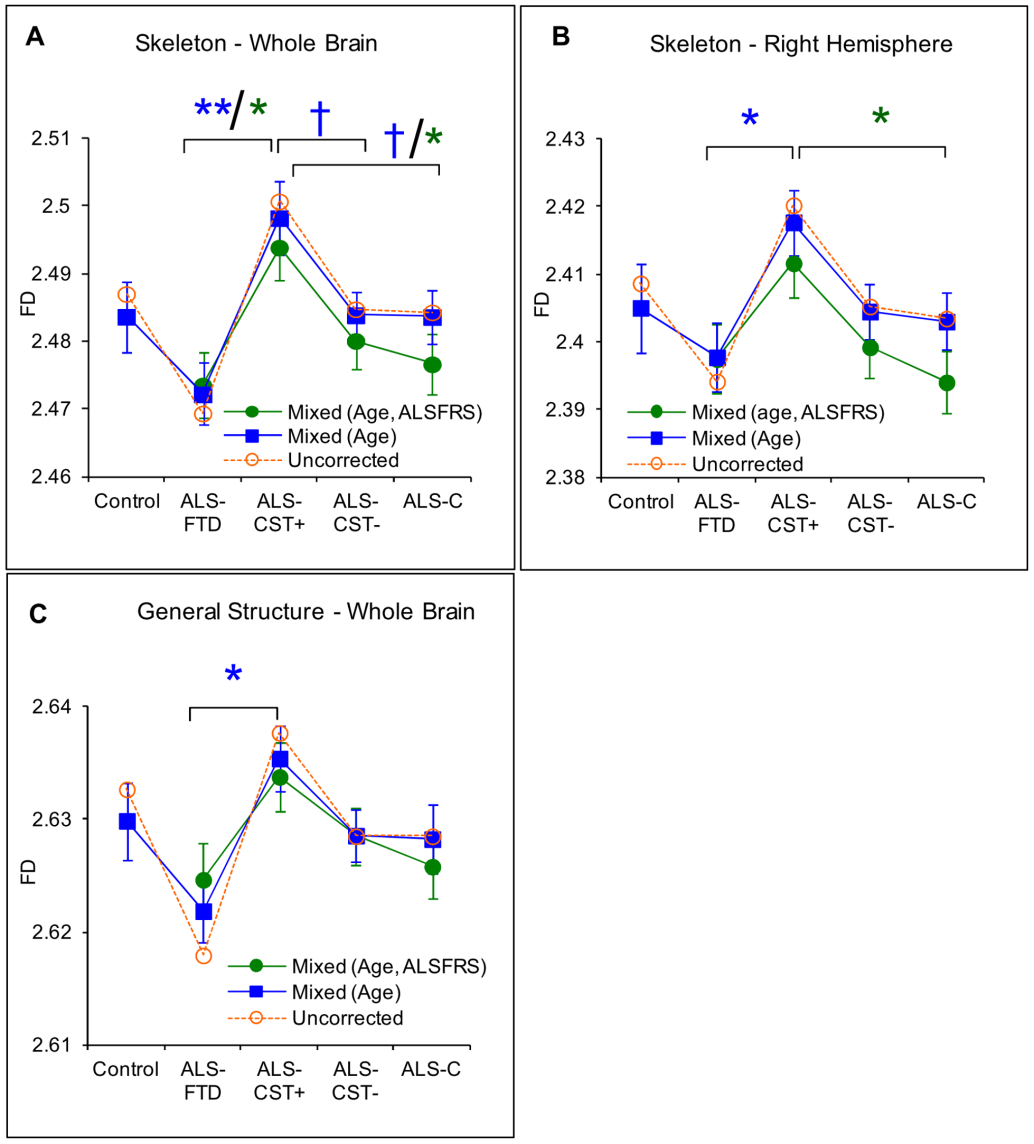
Between group comparison showing significant difference. Uncorrected means are shown in dashed line and corrected means (mixed model) and standard error of the mean are shown in solid line. Data in blue are for the mixed model with gender, age as covariates and in green with gender, age and ALSFRS-R as covariates. Corrected mean comparison between groups was performed using the Tukey multiple comparison method. † p<0.1,* p<0.05, ** p<0.001 (A) skeleton-whole brain, (B) skeleton right hemisphere, and (C) general structure whole brain.

Significant age effect was found only on whole brain general structure FD values (Fig. 1). Overall a decreasing trend in FD values with age was observed indicating that the WM structural complexity and WM volume decrease with age which is consistent with findings from Zhang et al [3].

**Figure 2:**
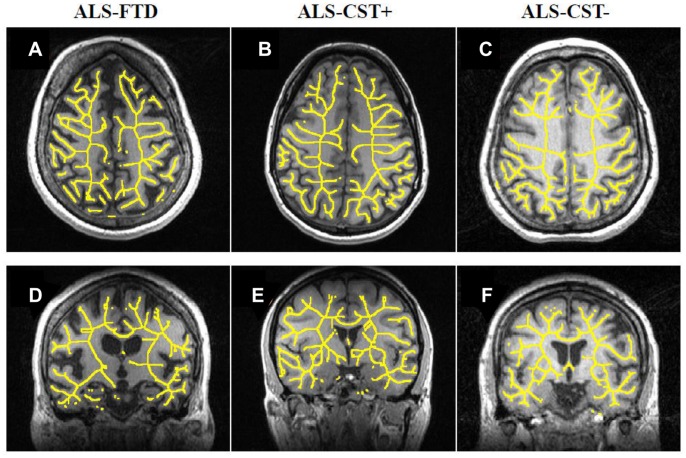
Typical illustration of 2D WM skeleton superimposed on an anatomical T1-weighted images. WM skeleton complexity is reduced in an ALS-CST– patient (C) axial view and F) coronal view) and an ALS-FTD patient (A) axial view and D) coronal view) compared to an ALS CST+ patient (B) axial view and E) coronal view).

**Figure 3:**
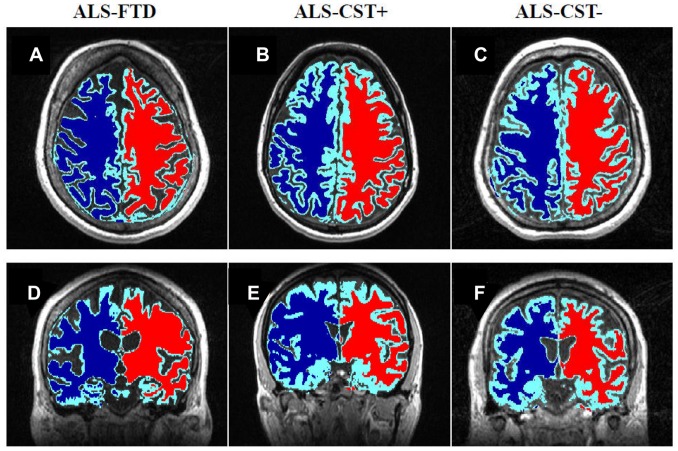
Typical illustration of reduced WM general structure complexity. WM general structure complexity in an ALS-FTD ((A) axial view and (D) coronal view), and an ALS-CST– ((C) axial view and (F) coronal view) patient compared to an ALS-CST+ patient ((B) axial view and (E) coronal view).

Post hoc analysis revealed that the corrected FD values of whole brain skeleton (*p* = 0.001) (Fig. 2A), right hemisphere skeleton (*p* = 0.03) (Fig. 2B), and whole brain general structure (*p* = 0.02) (Fig. 2C) were significantly reduced in ALS-FTD patients when compared with ALS-CST+ patients. Also there were a trend toward significance for whole brain skeleton FD values between ALS-CST+ and both ALS-CST– (p = 0.10) and ALS-Cl patients (p = 0.10). No significant differences in FD values were observed between control and ALS patients or within non-demented ALS subgroups. These results suggest that the shape complexity pattern in ALS-CST+ group (Fig. 2A, D, Fig. 3A, D) was obviously greater than that in ALS-FTD group (Fig. 2B, E, Fig. 3B, E), including the right hemisphere skeleton and whole brain skeleton and general structure. A 3D rendering of WM skeleton in a typical control and ALS patients from each subgroup is shown in the Figure S1.

**Figure 4:**
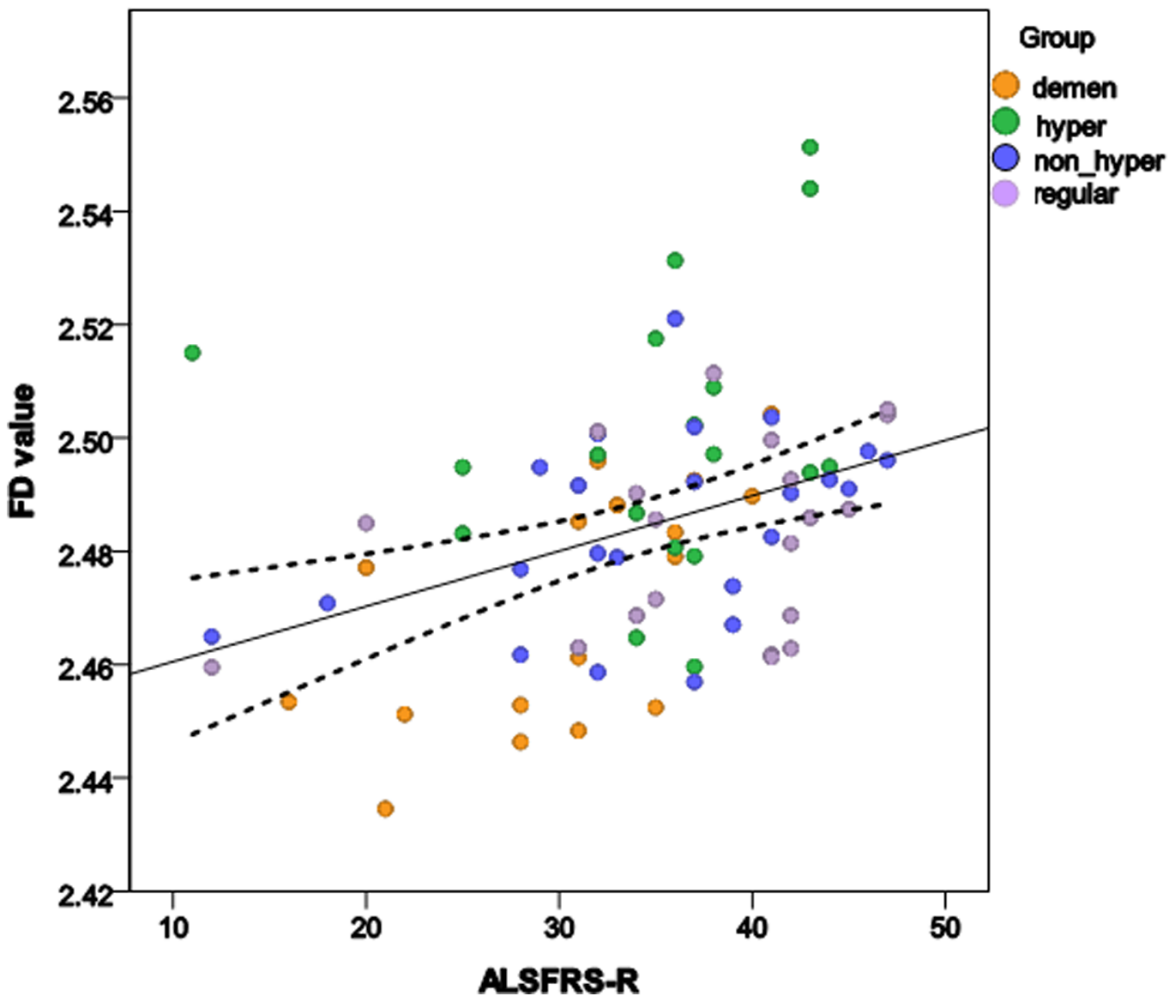
Correlation between FD values and ALSFRS-R score in ALS patients.

Significant correlations (positive) were observed between ALSFRS-R and FD values of WM skeleton, surface and general structure in whole brain, left and right hemispheres (Table 4, Figure 4), indicating that complexity level of the WM structures declines (FD values decreases) in ALS patients as their functional status worsens.

**Table 4:** Spearman’s rank correlation coefficient and significant *p* values between FD values of skeleton, surface and general structure in both hemispheres and in whole brain WM with ALSFRS-R score (corrected for multiple comparisons using FDR p<0.007).

	Rho (r)	*P* value
*Left Hemisphere WM*		
Skeleton	0.369	<0.001*
Surface	0.312	0.005*
General Structure	0.302	0.007
*Right Hemisphere WM*		
Skeleton	0.365	<0.001*
Surface	0.360	<0.001*
General Structure	0.229	0.04
*Whole brain WM*		
Skeleton	0.431	<0.001*
Surface	0.399	<0.001*
General Structure	0.369	0.001*

FD-fractal dimension, WM-white matter, FDR-false discovery rate, ALSFRS-R-ALS functional rate score-revised.

* P<0.005 (false discovery rate corrected p value).

No significant correlation was observed between FD values of general structure, skeleton or surface with disease duration and disease progression rate. The FD values also failed to reach significant difference between ALS patient populations with different El Escorial scores.

### Sensitivity Analysis

When performing the sensitivity analysis performed by including ALSFRS-R in the mixed model to rule out the possibility that the observed difference in FD was due to group differences in ALSFRS-R, the observed significant difference(s) in FD between ALS-FTD and ALS-CST+ disappeared for the whole brain general structure and the right hemisphere skeleton, but remained for the whole brain skeleton. Since this adjustment led to a decrease in FD for ALS-Cl, differences between ALS-CST+ and ALS-Cl became statistically significant for the right hemisphere and whole brain skeleton.

## Discussion

The main goal of this study was to investigate morphometric brain WM changes in ALS patients compared to controls and also among ALS patients with different clinical phenotypes using quantitative FD method and compare it with the common VBM approach. The ultimate goal would be to identify a potential biomarker for earlier and more accurate diagnosis of ALS. The main findings of this study are: 1) No significant difference in FD values was observed between control and any of the ALS subgroups, 2) However, FD values of WM skeleton and general structure were significantly different between ALS-CST+ and ALS-FTD groups i.e. the FD method was sensitive in identifying differences between ALS subgroups, shown in Figure 1 and Figure 2 (A,B,D &E), 3) In contrast, no significant WM volume changes were observed using VBM analysis and 4) significant correlation was observed between FD values and ALS functional disability score ALSFRS-R as shown in Figure 4, indicating that FD changes may reflect disease progression; future longitudinal studies may confirm this. These findings are discussed in detail in the following paragraphs.

### Participants’ Demographics and Patients’ Disease Characteristics

ALSFRS-R is a measure of daily functional activity impairment (e.g. speech, swallowing and walking) with lowest score showing the greatest impairment. Overall, ALSFRS-R showed little difference among groups suggesting that data for each ALS subgroup were collected at a similar disease stage in terms of functional impairment.

Significant group differences in disease duration among ALS subgroups were also observed. However given its arbitrary nature and the wide disparity in disease progression rate, this may not be a reliable measure of how far the disease has progressed. This may be a possible reason for its lack of significant correlation with FD. Similarly disease progression rate measures how fast rather than how far the disease has progressed, and it is therefore not surprising to find lack of correlations with FD. Disparity in disease progression rate among ALS subgroups suggests different pathophysiological mechanisms of the disease process.

### WM VBM Results

We failed to observe significant brain WM volume changes between controls and ALS patients or among the ALS subgroups using VBM approach. Chang et al [12] also failed to observe significant WM volume changes in ALS patients with or without dementia when compared to controls. Whereas, Ellis et al [11] observed significant WM volume reduction from precentral gyrus to internal capsule along the corticospinal tract in ALS patients with bulbar onset compared to those with limb onset, but failed to observe any difference between ALS patients pooled together and control. Similarly, Abrahams et al [10] observed significant WM volume reductions (compared to controls,) in frontotemporal locations in ALS patients with cognitive deficits measured by verbal fluency test. The discrepancy in VBM results between ours and other ALS studies could potentially be due to the following factors: (i) Ellis et al [11] used T2-weighted images, which provide poor contrast between GM and WM structures compared to the 3D high resolution T1-weighted images as used in this study; high GM-WM contrast is essential for robust tissue segmentation. (ii) Relatively thick slices (3 mm) in Abrahams et al [10] study as opposed to ours (1-mm thickness) might have contributed to inaccuracies in WM and GM segmentation from partial volume effects. (iii) Most previous VBM studies [10], [12] in ALS employed parametric GLM as opposed to the highly recommended non-parametric tests [29], [30]. We found that VBM results to be significantly different between parametric vs. non-parametric statistical models; specifically, more atrophied voxels were identified when non-parametric tests were employed compared to parametric GLM model (unpublished observation, manuscript under review), indicating that using different (sometimes inappropriate) statistical methods can result in spurious results.

Absence of significant changes in WM volume between controls and ALS patients in our study could be due to the fact that most of our ALS patients were in the early disease stage, i.e., a majority of them had an EES score below 3. This most likely implies that structural changes occur at the microscopic level (we observed significant differences in DTI metrics between control and ALS subjects) undetectable by the VBM method which is more sensitive to macroscopic level changes. An alternative explanation would be that VBM analysis is not sufficiently sensitive to detect the specific macroscopic changes occurring in ALS. The highly stringent statistical significance level imposed on the VBM method to compensate for multiple comparisons make it also more difficult to reach significance and observe an effect. In addition to axonal and myelin degeneration, gliosis is also widely reported in ALS [31], [32], which may not lead to the level of macroscopic volume changes detectable with VBM. Absence of any significant correlation between WM volume and clinical measures further supports this view.

### Fractal Dimension (FD) Results

FD approach differs from VBM, in which it quantitatively assesses shape or morphometric features, i.e., complexity level of the WM structure. No significant differences in FD values in any of the estimated WM features (surface, skeleton or general structure) were observed between control and ALS patients. A reason for this is some of the control subjects were not true healthy controls as some of them had non-ALS neurological disorders, such as Parkinson’s disease and chronic headache; this might have affected the measured FD values and eventually the results of comparisons with the patient groups. Among ALS subgroups FD values reached significance only between ALS-CST+ and ALS-FTD groups in general structure, right hemisphere skeleton and skeleton of the whole brain. ALS-CST+ group had highest FD values in all shape features (surface, skeleton and general structure) compared to other ALS subgroups and even to controls, even though statistical significance was not always reached after correcting for multiple comparisons. ALS-FTD on the other hand had the lowest FD values; whereas, ALS-Cl and ALS-CST– had similar FD values. However, a possible reason for not always reaching statistical significance could be that we have opted a conservative statistical significance threshold by correcting for multiple comparisons. However, given that the number of significant occurrences before correction for the right hemisphere skeleton (3 occurrences) and for whole brain skeleton (5 occurrences) were much greater than if this was due to chance (which would have been 1 out of 20 at p = 0.05), those observed differences are most likely not false positives. Under this consideration, when not correcting for multiple comparisons, FD of the whole brain skeleton, in ALS-CST+ was found to be significantly greater than all other groups (even after including ALSFRS-R as a covariate in the model), whereas FD of the ALS-FTD group was consistently lower than all other groups (but it was only lower than ALS-CST+ when ALSFRS-R was included as a covariate). Similarly, for the right hemisphere skeleton, FD for ALS-CST+ was found to be significantly greater than all other ALS subgroups (but not controls) even after including ALSFRS-R as a covariate. The significant difference in FD values between ALS-CST+ and ALS-FTD groups and other ALS subgroups suggest a different neurodegenerative process for these two groups of ALS patients.

The greatest FD contrast was observed for the whole brain skeleton. Because FD measures of the WM skeleton represent complexity level of the WM fiber bundle network (e.g. fibers crossings and bifurcations), the decreased skeleton FD value in ALS-FTD patients (when compared to all other ALS subgroups) may indicate diminished connectivity in the WM network. It has been previously reported that gray matter (GM) loss along with abnormal diffusivity in WM tracts connecting the afflicted GM regions contributed to the damage of neuronal network in the WM, particularly in the frontal and temporal lobes in dementia [33]. This is highly consistent with what we observed in ALS-FTD patients: using GM VBM and cortical thickness analyses, we found significant GM volume atrophy and reduction in cortical thickness in frontal and temporal lobes in ALS-FTD patients (same population as in this study) compared to control and other ALS subgroups (manuscript under review). In addition, GM atrophy and cortical thickness changes were widespread and also affected both motor and extra-motor regions implicated in ALS. This finding and Whitwell et al. [33] observations linking GM loss, WM abnormalities and dementia suggest that the significant reduction in brain WM interior structure complexity level in ALS-FTD patients may be a consequence of GM loss as a result of dementia or vice versa. In contrast to ALS-CST+, these also suggests widespread GM and WM degeneration in ALS-FTD.

Also, diffusion tensor imaging (DTI) studies in ALS showed that mean diffusivity remained unaffected in the CST in ALS patients even though a decrease in FA was reported [11], [34]. This was attributed to the fact that due to gliosis, total obstruction to water molecule may remain unchanged so no difference in mean diffusivity values were observed between control and ALS, whereas reduction in fractional anisotropy may be due to axonal and myelin degeneration. It is not clear yet how WM FD as a metric characterizing structural complexity at a macroscopic level is affected by these different neurodegenerative processes such as demyelination, Wallerian degeneration, axonal degeneration and gliosis. It might be hypothesized that continual progressive degenerations at the microstructures may over time manifest at the macrostructures such as small fiber tracts disappearance eventually affecting the complexity level of the WM structure. Matsusue et al [32] who correlated changes in T2-weighted images with histophathological changes in ALS patients with dementia reported not only gliosis but also T2 hyperintensities in temporal lobes and mild CST degeneration but no CST atrophy. Similarly in ALS-CST+ patients, the hyperintense T2 signal widely reported along CST could be due to gliosis, edema, axonal degeneration, and acute demyelination [35].

Differences in WM FD values and previously observed GM changes, as well marked clinical features differences between ALS-CST+ and ALS-FTD groups indicate that the mechanism of neurodegeneration may be different between these two groups of ALS patients. For ALS-CST+, the lack of observed GM loss and higher FDs likely suggests “axonopathy”. We speculate that the reason for higher FD values seen in ALS-CST+ could be due to cell inflammation and movement of other cells like macrophages. Future studies correlating FD estimates with histopathology should support our observation.

In general FD values in ALS-CST+ group were found to be elevated when compared to other ALS subgroups and even to control while loss of complexity may be expected with degeneration. Studies in multiple sclerosis [16], [36] may provide an explanation for the underlying physiological mechanism for the abnormally high FD in ALS-CST+ and low FD in ALS-FTD. They proposed that the reduction in WM FD could be due to increased water content, decreased myelin content and other inflammatory events leading to more amorphous tissue [16]. In contrast, they suggest that the increase in GM complexity, they observed in multiple sclerosis population compared to controls [36] might be due to inflammatory component (i.e. microglia activation) and cellular changes (synapse pruning, demyelination, blood-brain barrier changes etc). Future studies combining histopathological correlations with FD changes should reveal more about the underlying physiological changes due to ALS disease process.

We failed to observe significant alterations in surface FD whereas Esteban et al [16] in multiple sclerosis did and attributed this to juxtacortical WM lesions and grey matter abnormalities as surface FD values were calculated on the WM-GM boundary voxels. We observed significant changes in the right hemisphere WM skeleton which probably reflects changes in WM shape, complexity and interior cerebral lesion. Possible reasons for this include that neurodegenerative changes may not have been picked by the fewer surface voxels in surface FD estimates. On the other hand, the failure of general structure to show any regional FD change, may be due to the lack of specificity associated with the inclusion of the whole brain.

The positive relationship between FDs (skeleton FD, surface FD and general structure FD) and ALSFRS-R scores suggests that diminished complexity of the interior structure, sulcal widening and WM atrophy all contribute to the disability of ALS patients. Diminished complexity of the WM interior structure reflects a simpler WM network, in which some fiber bundles, bundle crossings and bifurcations might be lost. A positive correlation between the fractional anisotropy of the CST and ALSFRS-R score has been found previously [37], suggesting that a low ALSFRS-R score is associated with loss of fiber connectivity and axonal degeneration. Sulcal widening reflects reduced brain WM outer surface and GM inner surface. It may represent the death or atrophy of neural cell bodies.

A possible reason why VBM analysis failed to reveal similar kind of changes as FD could be due to the fact that VBM looks only at gross changes i.e. including both shape and other amorphous structural changes, whereas FD is more sensitive/specific to shape. Since our patient population happened to be in the early disease stage and in whom we observed FD changes (but not VBM changes) we believe that during early stages, ALS disease process may affect WM shape first (feature of the morphometry) before affecting other morphometric features.

To our knowledge this is the first study in ALS to quantify shape morphometry and to compare VBM with FD approaches in detecting brain WM structural degeneration among different clinical phenotypes of ALS. It is important to bear in mind that small differences in FD values between groups (see Table 2) reflect relatively big changes in their shape complexity as FD is measured in log scale. Future studies combining FD with diffusion tensor imaging and histopathological correlations would shed more light on the mechanisms of neurodegeneration in ALS and the potential role of the FD measure as a biomarker of ALS and disease progression. Some of the limitations of our study are: a) some of the control subjects were not true healthy controls as some of them had non-ALS neurological disorders, such as Parkinson’s disease and chronic headache; this might have affected the measured FD values and eventually the results of comparisons with the patient groups. b) Significant differences in age and ALSFRS score between ALS subgroups may have contributed to the observed difference in FD. Our study in aging showed significantly higher FD values in young than elderly subjects [3]. However, care was taken to control for these cofounders, which in almost all cases led to a more conservative estimate of the group differences. However there are inherent limitations with this a posteriori adjustment [38]. Future studies should attempt to recruit age and ALSFRS matched individuals to confirm the observed difference between groups. c) Absence of longitudinal evaluations of brain GM and WM structures and their function, and their relation with cognitive and sensorimotor performance of ALS patients. Such longitudinal studies would provide critical information for understanding the disease progression and its underlying neural mechanisms, and for seeking effective treatments.

## Conclusion

ALS patients with frontotemporal lobe dementia have greatest brain white matter structural degeneration among ALS patients with different clinical signs, measured by FD that estimates complexity level of an object and brain WM structure in this study. Our results indicate that the FD is a more sensitive index of brain WM structural integrity than volumetric measurement in ALS population. Grey matter loss in the frontal and temporal lobes could be the primary cause of the WM degeneration in this group of patients. The level of WM structure degeneration in ALS is phenotype dependent given the higher complexity level in patients with ALS-CST+ and lower complexity level in patients with ALS-FTD. FD measurement of WM structural complexity correlates significantly with widely used clinical score of ALSFRS-R, suggesting that the structural measurement reflects functionality of the patients. Among the three WM shape representations measured and based on their correlations with clinical evaluations, the WM skeleton seems to be the most sensitive substructure for detecting WM structural degeneration in ALS patients.

## Supporting Information

Figure S1 **3D rendering of WM skeleton image in a typical.** A) Control subject, B) ALS patient with dementia, C) ALS patient with CST hyperintensity and D) ALS patient without CST hyperintensity.
(TIFF)
