# Supplementary figures and images for: Brain White Matter Shape Changes in Amyotrophic Lateral Sclerosis (ALS): A Fractal Dimension Study
Source: PLoS One. 2013 Sep 9;8(9):e73614. doi: 10.1371/journal.pone.0073614 (PMC3767806; doi:10.1371/journal.pone.0073614)

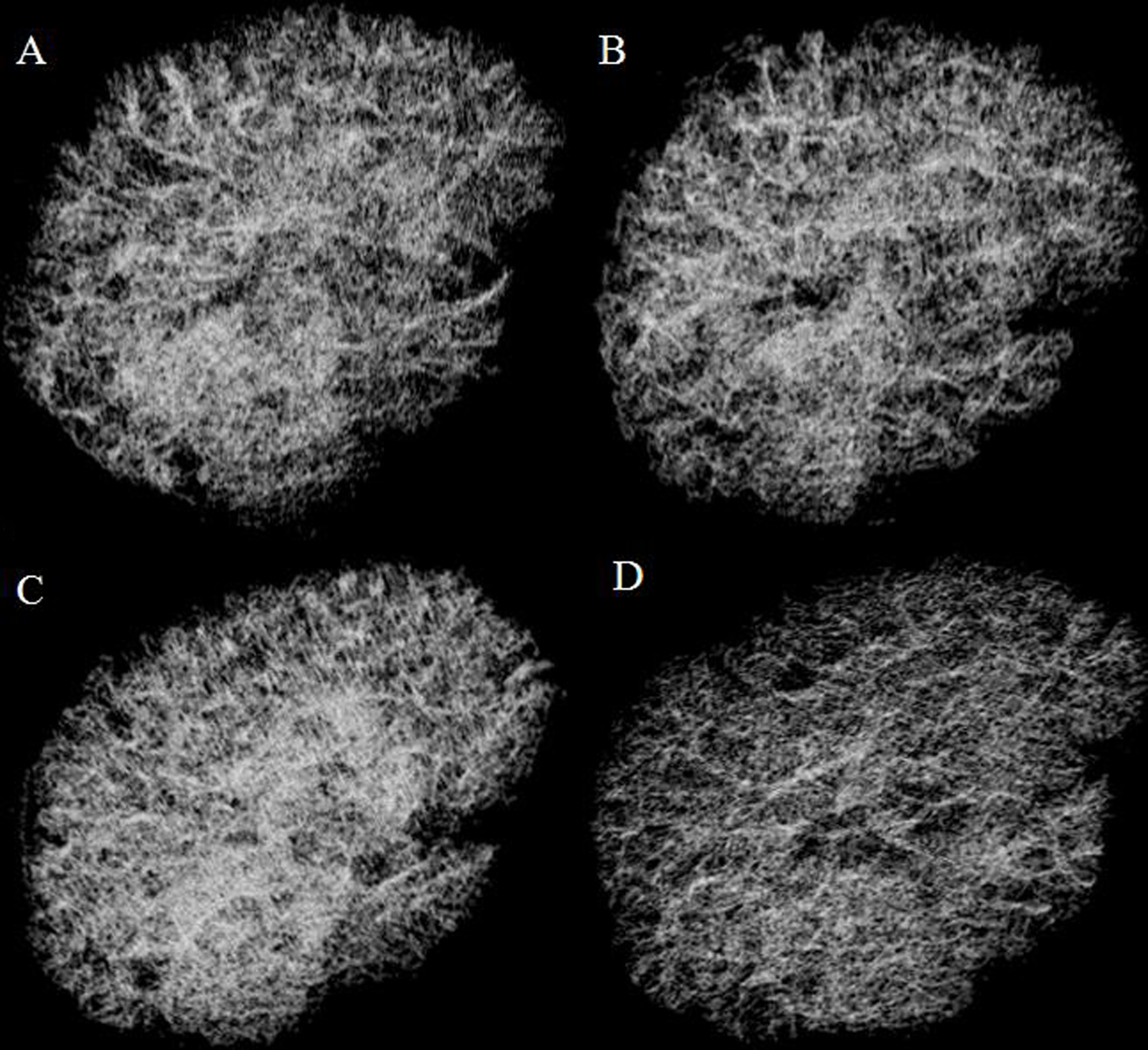

Supplement: Figure S1 — 3D rendering of WM skeleton image in a typical. A) Control subject, B) ALS patient with dementia, C) ALS patient with CST hyperintensity and D) ALS patient without CST hyperintensity. (TIFF) [file pone.0073614.s001.tiff]
